# Optimized Brain Extraction for Pathological Brains (optiBET)

**DOI:** 10.1371/journal.pone.0115551

**Published:** 2014-12-16

**Authors:** Evan S. Lutkenhoff, Matthew Rosenberg, Jeffrey Chiang, Kunyu Zhang, John D. Pickard, Adrian M. Owen, Martin M. Monti

**Affiliations:** 1 Department of Psychology, University of California Los Angeles, Los Angeles, California, United States of America; 2 Division of Neurosurgery, University of Cambridge, Addenbrooke’s Hospital, Cambridge, United Kingdom; 3 Brain & Mind Institute, Natural Science Building, The University of Western Ontario, London, Ontario, Canada; 4 Brain Injury Research Center (BIRC), Department of Neurosurgery, Ronald Reagan Medical Center, University of California Los Angeles, Los Angeles, California, United States of America; Beijing Normal University, Beijing 100875, China

## Abstract

The study of structural and functional magnetic resonance imaging data has greatly benefitted from the development of sophisticated and efficient algorithms aimed at automating and optimizing the analysis of brain data. We address, in the context of the segmentation of brain from non-brain tissue (i.e., brain extraction, also known as skull-stripping), the tension between the increased theoretical and clinical interest in patient data, and the difficulty of conventional algorithms to function optimally in the presence of gross brain pathology. Indeed, because of the reliance of many algorithms on priors derived from healthy volunteers, images with gross pathology can severely affect their ability to correctly trace the boundaries between brain and non-brain tissue, potentially biasing subsequent analysis. We describe and make available an optimized brain extraction script for the pathological brain (optiBET) robust to the presence of pathology. Rather than attempting to trace the boundary between tissues, optiBET performs brain extraction by (i) calculating an initial approximate brain extraction; (ii) employing linear and non-linear registration to project the approximate extraction into the MNI template space; (iii) back-projecting a standard brain-only mask from template space to the subject’s original space; and (iv) employing the back-projected brain-only mask to mask-out non-brain tissue. The script results in up to 94% improvement of the quality of extractions over those obtained with conventional software across a large set of severely pathological brains. Since optiBET makes use of freely available algorithms included in FSL, it should be readily employable by anyone having access to such tools.

## Introduction

In the past 20 years, magnetic resonance imaging (MRI) has gained a prominent role in the study of brain structure and function in healthy and pathological brains. Following the increased availability and popularity of this technique, a number of software tools have been developed and widely adopted, to automate the treatment and analysis of brain MRI data, including Statistical Parametric Mapping (SPM; [1]), Analysis of Functional NeuroImages (AFNI; [2]), FMRIB’s Software Library (FSL; [3]), and Brain Voyager ([4]), among others. The efficacy and efficiency of these tools is affirmed by the fact that virtually all the literature published in this domain relies in large part on these available software packages for several kinds of data analysis. Nonetheless, there are some circumstances in which these tools encounter difficulties which might result in suboptimal performance and risk biasing results (see also [5]). One such circumstance is the study of function and structure in severely pathological brains.

Clinical research has greatly benefitted from neuroimaging techniques across a variety of domains, including neurodegenerative disorders (e.g., Alzheimer’s disease, Parkinson’s disease), mental illnesses (e.g., schizophrenia, depression), and brain injury. The presence of severe pathology, however, is often very challenging for standard analysis algorithms making them prone to suboptimal performance and at risk of introducing small but systematic differences across groups, particularly in the common situation in which healthy individuals are compared to patient cohorts. The present research addresses this problem in the domain of the severe pathology associated with vegetative and minimally conscious states [6], a field that has witnessed, in the last 15 years, an exponential growth thanks to functional and structural MRI [7]. In this report, we focus on the challenges posed by this population to the process of brain extraction, which is to say the separation of brain from non-brain tissue (including the skull, neck, eyes, etc.). These difficulties are principally due to the fact that the performance of many skull-stripping algorithms is based on Bayesian priors derived from the healthy brain (e.g., FSL’s Brain Extraction Tool; BET, [8]; AFNI’s 3dSkullStrip, [2]) which are not representative of severely damaged brains and can thus perform sub-optimally in the presence of gross pathology. Indeed, in patients with abnormal brain morphology, brain image outlines are often so distorted that the automated segmentation tools incorrectly classify tissues and subsequently overestimate or underestimate brain volume. As a result, structural analysis of local or global brain shape and volume, tractography, brain registration, as well as functional localization in ‘standard’ space may result in biased estimates.

As described below, we develop, test, and make available a novel, automated and optimized brain extraction script for pathological brains (optiBET). This script combines freely available and widely employed MRI software tools into an optimized workflow that achieves high-quality brain extraction even in the presence of severe and gross pathology. Our methodology is based on the calculation of an initial approximate brain-extraction, linear and nonlinear projections from the individual subject space to the MNI template space, and, finally, back-projection of an MNI brain-only mask into the original subject space which is then used to mask-out non-brain tissue from the original image. To optimize our script, we varied and combined a number of parameters including (i) the specific algorithm and options employed to perform the initial approximate brain extraction, (ii) the algorithm used to project the initial extraction into the MNI template reference space, and (iii) the mask which underwent back-projection to the subject’s original space (chosen among the several standard masks available within FSL). Results obtained from each tested combinations of these three steps were compared against manually-traced brain-only benchmark masks. Our analyses show that the optiBET script results in substantial improvement in brain extraction over all other tested tools, across a large set of brain images obtained from a cohort of 74 patients presenting gross brain pathologies due to severe traumatic and non-traumatic brain injury.

## Methods

### Subjects

Data from seventy-four patients with disorders of consciousness were obtained as part of a large neuroimaging study of severe traumatic brain injury (TBI). Subsets of these data have been previously described, in the context of functional and structural studies, in several previous publications; e.g., [9], [10], [11], [12], [13], [14]. Procedures for this study were approved by the Cambridge Local Regulatory Ethical Committee (LREC). For each patient, written consent was given by their legal representative.

### MRI Data Acquisition

T1-weighted three-dimensional magnetization-prepared rapid acquisition with gradient echo images (MP-RAGE; [15]) were acquired on a 3T Siemens Tim Trio MRI machine at the Wolfson Brain Imaging Center (WBIC), Addenbrookes Hospital, in Cambridge UK (TR = 2,300 ms, TE = 2.47 ms, TI = 900 ms, 150 slices, 1×1×1.2 mm resolution).

*Brain extraction work-flow.* The optiBET script is conceived as a 4-step process which, rather than attempting to outline the brain-to-skull boundary, a task that is extremely challenging for all conventional algorithms when applied to pathological brains, performs brain extraction by back-projecting a standard brain-only mask into the individual subject’s space to then mask-out all the tissue falling outside of it. The 4 basic steps of the optiBET script are (i) initial approximate brain extraction; (ii) projection of the approximate extraction mask from subject space to MNI template space; (iii) back-projection of a brain-only mask – taken from the standard mask database freely available within FSL – from the standard MNI template space to the original subject’s image (via the inverse of the transformation matrix obtained in step ii); and (iv) mask-out from the patient’s whole head T1 image (in native space) of all tissues not included in the back-projected standard brain-only mask. The masking procedure is effectively a multiplication of the original head-only image by the binary back-projected brain only mask. (We include, as S1 Appendix, an annotated excerpt of the optiBET script which features a short explanation of each step, and the full citation – where available – or link for each routine employed in the tool. The full, open-source, optiBET script is freely available for download at http://montilab.psych.ucla.edu/fmri-wiki).

### Brain extraction optimization and evaluation

In order to optimize the above procedure, we compared, for steps i through iii, several different implementations (henceforth referred to as “pipelines”; all of which are based on routines that are freely available as part of existing MRI analysis packages). The initial brain extraction (step i) was attempted using three algorithms, each with and without a number of options. Brain Extraction Tool, part of the FSL software package, (BET; [8]) was implemented with and without image bias and residual neck reduction (‘B’), fractional intensity threshold (‘f = 0.1’) (as recommended by [5]), as well as robust estimation of the center-of-gravity (‘R’) and residual eye and optic nerve reduction (‘S’). 3dSkullStrip, part of the AFNI software package, (which is itself a modification of BET; [2]) was implemented with and without the ‘use skull’ option. Finally, we also employed the less common Robust Extraction software (Robex; [16]). The projection of the initial brain extraction from native image space into standard MNI template space (step ii) was performed either with a linear registration alone (using FLIRT with 12 degrees of freedom; [17], [18]), or by supplementing the linear transformation with a non-linear transformation (using FNIRT, also part of the FSL suite; see http://fsl.fmrib.ox.ac.uk/fsl/fslwiki/FNIRT). Back-projection of a brain-only standard mask (step iii) was attempted using a number of masks (N = 12) all freely available as part of the FSL suite and varying in resolution and method of calculation (avg152T1 brain tissueprior, avg152T1 brain, MNI152 T1 1 mm brain mask dil, MNI152 T1 1 mm brain mask, MNI152lin T1 1 mm brain, MNI152 T1 1 mm first brain mask, MNI152 T1 2 mm brain mask dil, MNI152 T1 2 mm brain mask dil1, MNI152 T1 2 mm brain mask, MNI152 T1 2 mm strucseg periph, MNI152 T1 2 mm strucseg, MNI152lin T1 2 mm brain mask). The back-projection itself was carried out by inverting the transformation matrix calculated in step ii.

In order to evaluate the quality of the extractions obtained with each pipeline, we compared their output to manually traced “benchmark” brain extractions (one per each of the 74 images employed). The difference between the benchmark and the automated extractions was calculated using a least squares approach in which the difference in intensity at each voxel between two (binarized) images (i.e., the benchmark and each automated extraction) was squared and then summed over all voxels in the image. This approach treats equally mismatches due to excessive erosion of brain tissues (i.e., overly conservative extractions) as well as inclusion of non-brain tissues (i.e., overly lenient extractions). In what follows, we use the median sum of squares as the measure of central tendency of each pipeline’s extraction quality because the distributions of mismatch values were significantly non-normal (as assessed by the Shapiro-Francia test; p<0.002 for each pipeline).

## Results

### Optimization results

**Figure 1:**
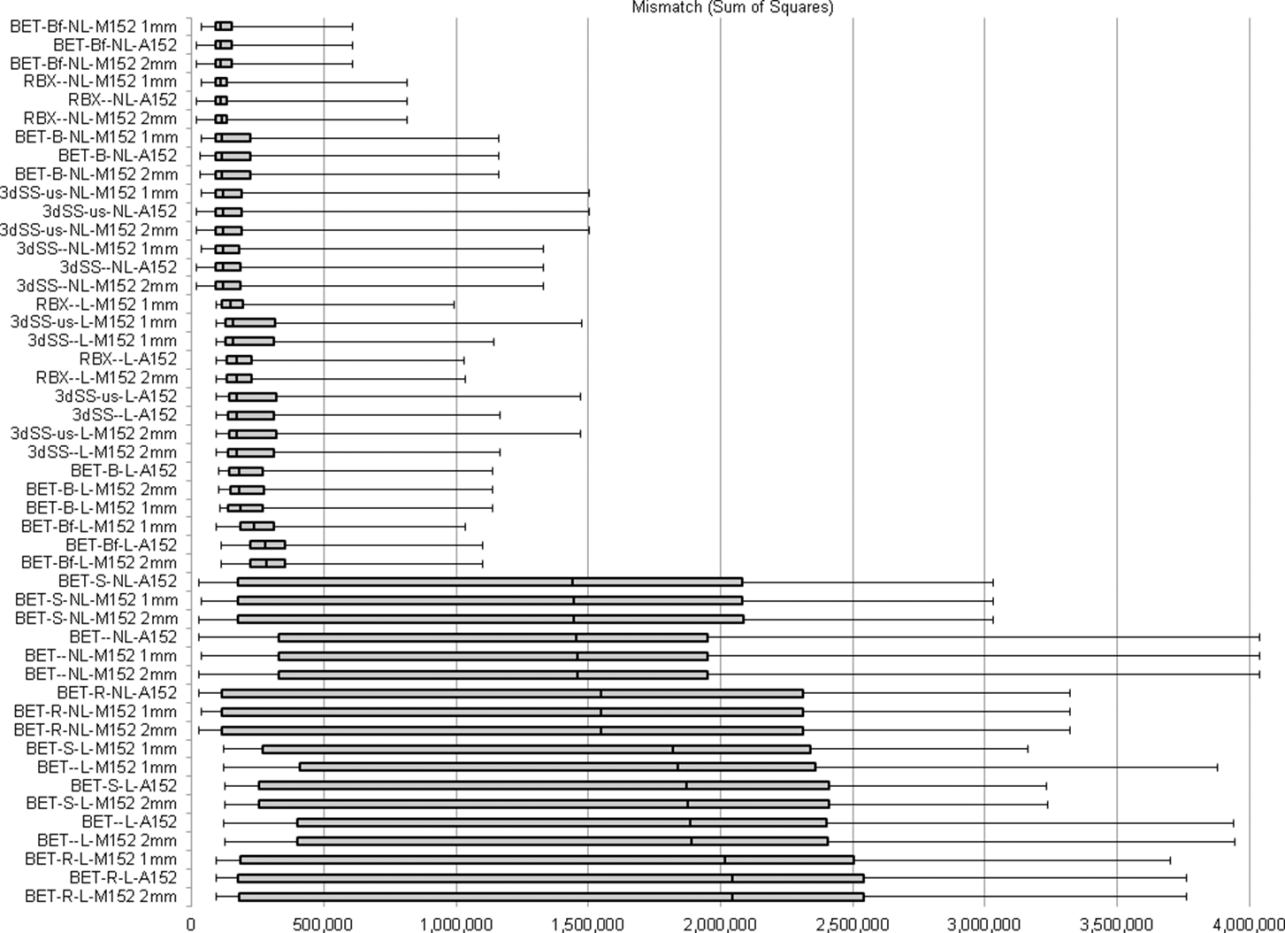
Optimization results. Box plot depiction of the mismatch between each tested pipeline and the manually brain-extracted “benchmark” images. Increased sum of squares implies lower extraction quality – as compared to the benchmark extractions. (RBX: Robex; 3dSS: 3dSkullStrip; us: useskull; L: linear transformation; NL: non-linear transformation; M152: MNI152; A152: Average152. The boxplot depicts the 25^th^, 50^th^ (i.e., median) and 75^th^ percentiles, and top and bottom whiskers depict the maximum and minimum values, respectively).

**Figure 2:**
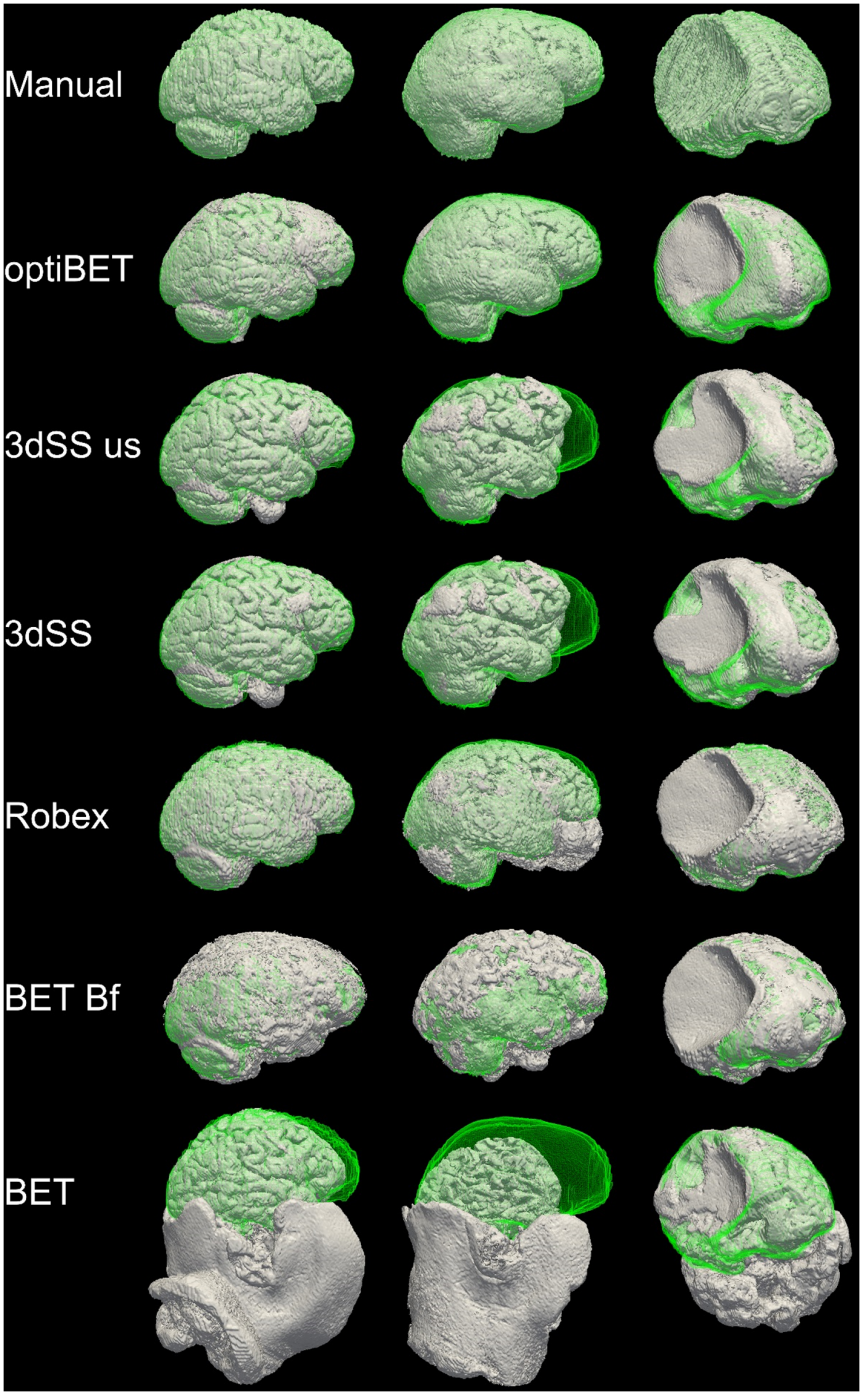
Sample extraction results. Rendering of brain extractions obtained from different algorithms/options (shown in grey) compared to the manually-traced benchmark (shown in green wireframe) for three sample patients with different degrees of brain pathology (little, medium, and high, for the left, middle and right columns respectively; see Fig. 3). The first row depicts the rendering of the benchmark volume (in grey) and the benchmark wireframe to illustrate a case of “perfect fit”. (Renderings obtained with ParaView [19]).

The results of our optimization process are reported (ranked by median extraction quality) in Table 1 and in Fig. 1. Overall, BET (with options ‘B’ and ‘f’) in combination with the sequential application of linear and non-linear transformations, resulted in the least mismatch with the manually traced extractions regardless of which mask was used for back-projection (i.e., step iii). Numerically, back-projection of the MNI152 T1 1 mm brain-only mask resulted in better quality extractions by 0.4% and 0.8%, as compared to the Average152 and the MNI152 2 mm masks, respectively. (We note that in Table 1 we only report results for the three mentioned masks since they were consistently better than all the other tested ones) As reported in Table 1, the best performing pipeline (BET ‘Bf’ with linear and non-linear registration and MNI152 1 mm mask back-projection) increased the extraction accuracy by as much as 94.7%, as compared to the worst performing pipeline (BET with option ‘R’ and linear transformation only), and as little as 1–2.4% and 3–5%, as compared to the second and third best set of pipelines (i.e., Robex with linear and non-linear registrations – for all back-projected masks – and BET with ‘B’ option, with linear and non-linear transformations – for all back-projected masks). Fig. 1, as well as in the last two columns of Table 1, gives some indication as to which conditions and procedures allow this approach to work. In particular, there are two points in the ranking of the pipelines’ performances where the quality of extraction exhibits a sudden degradation resulting in a sudden increase in sum of squares. One jump appears to be related to the specific combination of software and options employed for the initial approximate brain extraction (i.e., step i). Specifically, some initial extractions are sufficiently inaccurate that regardless of any subsequent processing (e.g., linear/non-linear registration and mask back-projection), it is not possible to obtain a high-quality final extraction. This issue is apparent for pipelines employing BET with options ‘R’, ‘S’, or with no options (see, for example, the bottom row of Fig. 2). Indeed, even in the best of these pipelines (namely, BET with the ‘S’ option, linear and non-linear transformation, and back-projection of the Average152 mask) the mismatch sum of squares increases to 1,441,469, as compared to the 280,047 achieved by the pipeline immediately preceding it in the ranking (i.e., BET with options ‘B’ and ‘f’, linear transformation only and back-projection of the MNI152 2 mm mask). While the top ranked pipeline offers a substantial advantage over both these latter pipelines, the relative advantage jumps from 61% (as compared to the BET ‘B,f’ with linear only option) to 94.7% (as compared to the BET ‘S’ option).

**Table 1:** Optimization results ordered by median mismatch.

Algorithm	Opt	Transf	Mask	Mismatch (SS)	% acc ↑
BET	Bf	NL	M152 1 mm	108,506.00	.
BET	Bf	NL	A152	108,947.50	0.41%
BET	Bf	NL	M152 2 mm	109,397.00	0.81%
RBX	.	NL	M152 1 mm	109,660.50	1.05%
RBX	.	NL	A152	110,626.50	1.92%
RBX	.	NL	M152 2 mm	111,157.50	2.39%
BET	B	NL	M152 1 mm	113,393.00	4.31%
BET	B	NL	A152	114,210.00	4.99%
BET	B	NL	M152 2 mm	114,542.50	5.27%
3dSS	us	NL	M152 1 mm	115,452.00	6.02%
3dSS	us	NL	A152	116,407.00	6.79%
3dSS	us	NL	M152 2 mm	116,654.00	6.98%
3dSS	.	NL	M152 1 mm	119,013.50	8.83%
3dSS	.	NL	A152	119,082.50	8.88%
3dSS	.	NL	M152 2 mm	119,337.00	9.08%
RBX	.	L	M152 1 mm	145,342.50	25.34%
3dSS	us	L	M152 1 mm	154,806.00	29.91%
3dSS	.	L	M152 1 mm	156,422.50	30.63%
RBX	.	L	A152	168,392.50	35.56%
RBX	.	L	M152 2 mm	168,983.00	35.79%
3dSS	us	L	A152	169,736.50	36.07%
3dSS	.	L	A152	170,308.00	36.29%
3dSS	us	L	M152 2 mm	170,364.50	36.31%
3dSS	.	L	M152 2 mm	170,841.00	36.49%
BET	B	L	A152	177,683.00	38.93%
BET	B	L	M152 2 mm	178,421.00	39.19%
BET	B	L	M152 1 mm	182,033.00	40.39%
BET	Bf	L	M152 1 mm	235,723.00	53.97%
BET	Bf	L	A152	279,380.00	61.16%
BET	Bf	L	M152 2 mm	280,047.00	61.25%
BET	S	NL	A152	1,441,469.00	92.47%
BET	S	NL	M152 1 mm	1,441,791.00	92.47%
BET	S	NL	M152 2 mm	1,442,392.00	92.48%
BET	.	NL	A152	1,455,430.50	92.54%
BET	.	NL	M152 1 mm	1,456,206.50	92.55%
BET	.	NL	M152 2 mm	1,456,223.00	92.55%
BET	R	NL	A152	1,546,753.00	92.98%
BET	R	NL	M152 1 mm	1,546,921.50	92.99%
BET	R	NL	M152 2 mm	1,547,395.50	92.99%
BET	S	L	M152 1 mm	1,818,280.00	94.03%
BET	.	L	M152 1 mm	1,836,867.50	94.09%
BET	S	L	A152	1,872,161.00	94.20%
BET	S	L	M152 2 mm	1,873,378.50	94.21%
BET	.	L	A152	1,886,844.50	94.25%
BET	.	L	M152 2 mm	1,888,055.50	94.25%
BET	R	L	M152 1 mm	2,014,282.00	94.61%
BET	R	L	A152	2,045,059.00	94.69%
BET	R	L	M152 2 mm	2,045,418.50	94.70%

(Mismatch is expressed in sum of squares; ‘Opt.’ indicates which options were used for each algorithm – if any; ‘RBX:’ Robex; ‘3dSS:’ 3dSkullStrip; ‘us’ indicates ‘use-skull’ option; ‘.’ indicates no option employed; ‘NL’ indicates linear plus non-linear transformation; ‘L’ indicates linear transformation only; ‘% acc ↑’ indicates the percent increase in extraction accuracy afforded by the best pipeline as compared to each assessed pipeline; ‘M152’ indicates MNI152 brain-only mask; ‘A152’ indicates Average 152 mask).

The second discontinuity in the mismatch ranking appears to be related to the choice of transformation (i.e., linear versus non-linear) employed to project the initial approximate extraction into MNI template space (i.e., step ii). Indeed, for any combination of algorithm and options used in step i, the pipeline featuring linear and non-linear transformations was always superior to the pipeline employing the same step i configuration but followed by a linear transformation only. As showed in Table 1, the first switch from linear plus non-linear to linear transformation only is marked by an increase in sum of squares of approximately 30,000, with the relative advantage offered by the top-performing pipeline jumping from approximately 9% to 25%. In the bottom half of the table, the second switch from non-linear to linear only transformations is not equally marked by a sizeable increase in sum of squares. This is presumably because the quality of the initial extraction is already sufficiently low that the choice of transformation strategy for step ii has almost no effect on the final extraction quality.

To summarize the results thus far, our optimization shows that the highest extraction quality were achieved employing, BET (with options ‘B’ and ‘f = 0.1’ [5]) for the initial approximate extraction (step i), non-linear transformation initialized by the linear transformation matrix (step ii), and back-projection of the MNI152 T1 1 mm brain only mask (step iii; using the inverse of the transformation estimated in step ii). In the reminder of this paper, we will refer to this specific pipeline as optiBET.

### Comparison between optiBET and standard brain extraction processes

**Figure 3:**
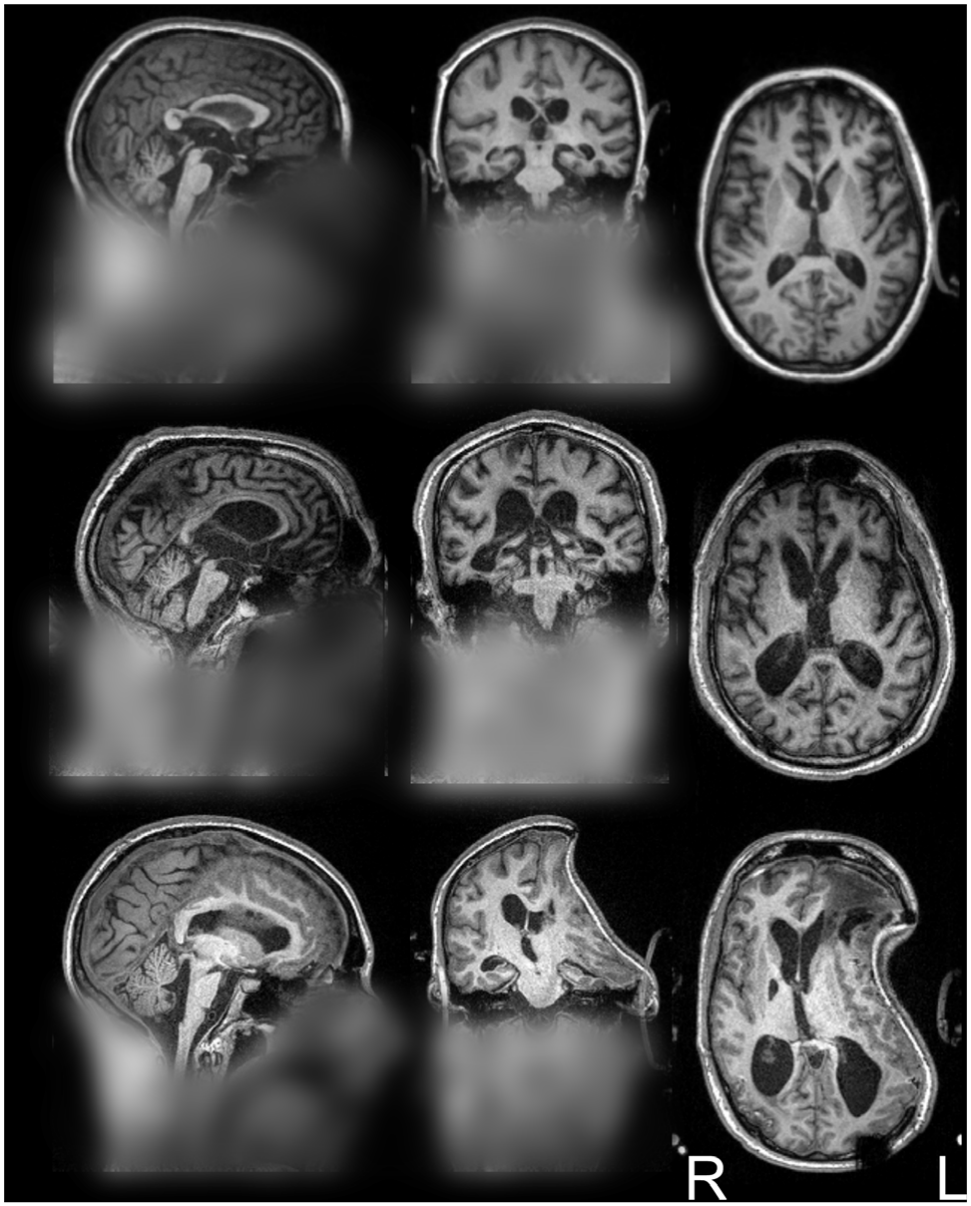
Sample patients’ full head images. Orthogonal projection view of the original full head images for the three patients employed in Fig. 2. From top to bottom the images exemplify patient with relatively low, medium, and high brain pathology – relative to this cohort of patients. (Images are displayed in radiological convention; blurring added to protect the privacy of the patients).

Table 2 reports the comparative performance, in terms of median extraction quality and mean computation time, between optiBET and other available brain-extraction tools (relative to the manually brain-extracted benchmarks). For a subset of these tools (marked by “*” in Table 2), we depict in Fig. 2 the fit of each extraction (in gray) to the benchmark (green wireframe) for three sample images with low, medium, and high brain pathology, respectively (see Fig. 3 for an standard 3-plane depiction of the wholehead image for each patient). In Fig. 2, areas where no green is visible indicate regions in which the brain extraction was overly permissive, thus failing to remove some non-brain tissue (e.g., row 5, column 2, in the figure). Areas where only the green wireframe is visible (e.g., rows 3 and 4, column 2), and no grey area is, indicate regions in which the brain extraction was overly conservative, removing brain tissue. As shown in Table 2, OptiBET exhibited the smallest mismatch with the manually-traced benchmarks. In terms of quality of extraction, Robex was a close second, followed by BET (with the ‘B’ and ‘f’ together, as well as ‘B’ alone), 3dSkullStrip (with and without the ‘use skull’ option), and, finally, the remaining configurations of BET, which all lagged by a very wide margin. As can be seen in the bottom row of Fig. 2, the poor performance of the “standard” BET (and the same is true of options ‘R’ and ‘S’) is likely attributed to a difficulty in establishing the center of the brain in images in which, as typical in the clinical domain, a sizeable section of the neck is also included. Indeed, use of the center-of-gravity option (c) is sufficient to fix the problem and thereby reduce the mismatch by approximately 73% (bringing the sum of squares from 1,527,457 to 414,079; it should be noted, however, that use of the ‘c’ option requires manual discovery of the center-of-gravity and input). As compared to the second best tool (i.e., Robex), OptiBET demonstrated an 8% reduction of mismatching voxels, while for all remaining algorithms and configurations, the mismatch reduction afforded by optiBET is at or above 50%, including 55% when compared to BET with options ‘B’ and ‘f’ (i.e., the configuration suggested in [5]), and 91% when compared to standard BET (i.e., with no options). In addition, optiBET also exhibited the lowest maximum mismatch value implying that the worst extraction performed by this script is better, by a 25% amelioration, than the worse extraction performed by other tools, with Robex being the second best performer. The ranking is switched when assessing the ‘best’ extraction, which is to say the extraction with the least mismatch.

**Table 2:** Relative performance summary (over all 74 subjects) for each brain extraction algorithm and tested option.

Algorithm	Opt	Mismatch (Sum of Squares)				Execution Time (MM:SS.MS)			
		Median	Min	Max	Δ (%)	Mean	Min	Max	Δ (%)
optiBET	.*	108,506	36,181	606,206	.	17∶33.6	14∶47.7	22∶32.2	.
ROBEX	.*	117,420	19,638	813,717	8%	01∶55.9	01∶33.4	02∶26.2	−89%
BET	B	215,439	94,234	1,121,534	50%	11∶05.5	08∶40.0	15∶11.4	−37%
	B,f*	261,263	117,738	1,019,837	58%	09∶02.1	08∶46.4	09∶15.5	−49%
	S	1,550,401	111,878	3,077,211	93%	04∶53.1	04∶28.1	05∶32.5	−72%
	.*	1,602,662	109,279	3,104,437	93%	00∶07.5	00∶06.7	00∶08.5	<−99%
	R	1,714,913	46,264	3,355,180	94%	00∶26.0	00∶22.2	00∶32.6	−98%
3dSkullStrip	.*	250,027	69,877	1,431,907	57%	02∶57.0	01∶00.1	04∶22.6	−83%
	us*	253,431	69,234	1,462,013	57%	03∶03.2	01∶08.3	04∶28.4	−88%

For extraction quality, we report the percent increase in quality afforded by optiBET, as compared to the other tools. For computation time, we report the percent reduction afforded by each tool, as compared to optiBET. (Unit for mismatch is sum of squares; units for execution time is minutes:seconds; ‘Opt.’ indicates which option configurations were used for each algorithm – if any; ‘us’ indicates ‘use-skull’ option; ‘.’ indicates no option employed; ‘*’ indicates the algorithm/option is depicted in Fig. 3).

The increased quality of brain extractions; however, comes at the cost of increased computation time. The last four columns of Table 2 report the average, minimum and maximum computation time for the three patients depicted in Figs. 2 and 3, as well as the time advantage (in %) of other tools as compared to optiBET. On average, our script runs a single brain-extraction in approximately 17 minutes. In comparison, BET with no options takes only 3 seconds – although, to the detriment of extraction quality. When looking at the top performing algorithms/configurations, however, Robex achieves high quality extraction in approximately 2 minutes, and the best BET configuration (with options ‘B’ and ‘f’ [17]) still takes only 9 minutes, that is, half the time of optiBET.

## Discussion

**Figure 4:**
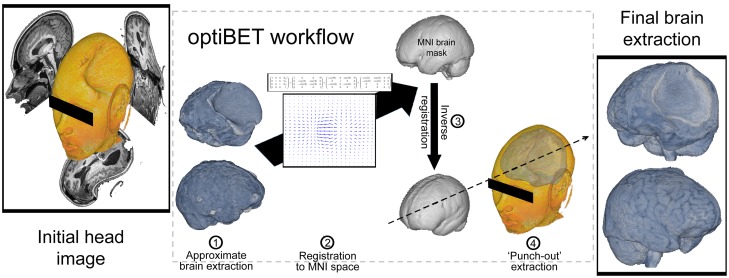
OptiBET workflow. Visual representation of the optiBET script workflow: (1) initial approximate brain extraction is performed using BET (and options ‘B’ and ‘f’, as suggested previously [5]); (2) sequential application of a linear and non-linear transformation from native space to MNI template space; (3) back-projection of a standard brain-only mask from MNI to native space; and (4) mask-out brain extraction.

In this paper we addressed the tension between the growing interest surrounding functional and structural MRI research in clinically relevant populations and the difficulty of standard algorithms to cope with severe brain pathology. In particular, we focused on brain extraction (also known as, skull-stripping), a process that is often performed at the very beginning of several different morphological and functional analyses. By combining freely available MRI software tools, and in particular those afforded by the FMRIB Software Library (FSL), and by using as a starting point settings previously shown to work in mild pathology [5], we were able to create an optimized workflow that, in severely pathological brains, improves brain extractions by a margin of 50% to 92%, as compared to commonly used algorithms available in FSL and AFNI (i.e., BET and 3dSkullStrip, respectively), and 8% as compared to the less commonly employed Robex. As summarized in Fig. 4, the increased quality of brain extractions was achieved by (i) creating a first approximate brain-extraction (performed with BET and options ‘B’ and ‘f’ [5], although the use of Robex in this step leads to very similar results), (ii) sequentially applying linear and non-linear transformations from native to MNI template space, (iii) back-projecting a 1 mm standard brain-only mask from MNI space back to native space (using the inverse transformation estimated in step iii), and finally (iv) masking the original full-head image with the back-projected standard mask. While our method proved to be the best in terms of quality of extraction, and also demonstrated to be the most robust method – with the least degradation of the extraction quality for the most pathological brains – it does come at the cost of increased computation time as compared to all other tools. In particular, the estimation of non-linear warp fields, as well as their inversion in order to back-project the brain-only mask, appear to be the major source of computation time (although, as demonstrated by our data, these two steps are also the reason high-quality brain extractions can be obtained even in severely pathological brains). Nonetheless, this is exclusively computer time, something that – given the continuously increasing availability of fast computer systems and standard use of scripting throughout MRI data analysis – is likely to be of minimal impact on data processing. Finally, while our tool resulted in large increase in image extraction quality for data containing severe pathologies affecting brain shape, standard brain-extraction tools might be regarded as more efficient (although equally effective) in the healthy brain.

## Supporting Information

S1 Appendix **Annotated optiBET code excerpt.** Excerpt of the main code, with annotations, showing step-by-step the flow of optiBET.
(DOCX)
