# Supplementary material for: Optimized Brain Extraction for Pathological Brains (optiBET)
Source: PLoS One. 2014 Dec 16;9(12):e115551. doi: 10.1371/journal.pone.0115551 (PMC4267825; doi:10.1371/journal.pone.0115551)
Supplement: S1 Appendix — Annotated optiBET code excerpt. Excerpt of the main code, with annotations, showing step-by-step the flow of optiBET. (DOCX) [file pone.0115551.s001.docx]

Lutkenhoff *et al* **Optimized brain extraction for pathological brains (optiBET)**

*Appendix: annotated optiBET code excerpt*

Excerpt of the main code, with annotations, showing step-by-step the flow of optiBET.

*Full open source code for optiBET is freely available at* [*http://montilab.psych.ucla.edu/fmri-wiki*](http://montilab.psych.ucla.edu/fmri-wiki)

#### 1. initial brain extraction (“step 1”) #########################

## Perform initial “approximate” brain extraction (use FSL unless the ## input specifies the AFNI option; this is referred to as “step 1” in ## the manuscript).

#

if [[ "$s1" == "bet" ]]; then

echo 1. BET -B -f 0.1 subject ${i} for initial extraction

bet ${iopt} ${i}_step1 -B -f 0.1

else

echo 1. AFNI 3dSkullStrip subject ${i} for initial extraction

$afnidir/3dSkullStrip -input ${iopt} -prefix ${i}_step1.nii.gz &>/dev/null

fi

######################################################################

REFERENCE: Smith SM (2002) Fast robust automated brain extraction. Human brain mapping 17: 143-155.

######################################################################

#### 2. Linear transform to MNI space (“step 2”) ####################

## Perform linear transformation of initial “approximate” extraction

## to MNI space (This is referred to as “step 2” in the manuscript).

#

echo 2. flirt subject ${i} to MNI space

flirt -ref ${FSLDIR}/data/standard/MNI152_T1_2mm_brain -in ${i}_step1.nii.gz -omat ${i}_step2.mat -out ${i}_step2 -searchrx -30 30 -searchry -30 30 -searchrz -30 30

######################################################################

REFERENCES:Jenkinson M, Bannister P, Brady M, Smith S (2002) Improved optimization for the robust and accurate linear registration and motion correction of brain images. NeuroImage 17: 825-841.

Jenkinson M, Smith S (2001) A global optimisation method for robust affine registration of brain images. Medical image analysis 5: 143-156.

######################################################################

#### 3. nonlinear transform to MNI space (“step 3”) ##################

## Follow the linear transformation with a non-linear transformation

## (Use the MNI152 2mm as default; This is referred to as “step 3” in ## the manuscript).

#

echo 3. fnirt subject ${i} to MNI space

fnirt --in=${i} --aff=${i}_step2.mat --cout=${i}_step3 --config=T1_2_MNI152_2mm

######################################################################

LINK: <http://fsl.fmrib.ox.ac.uk/fsl/fslwiki/FNIRT>

######################################################################

#### 4. QC: Generate image for QC ####################################

## This is a quality control step that generates an image of the

## original subject structural after transformation to MNI space

## which can (and should) be checked by the user.

#

echo 4. applywarp to put subject ${i} in MNI space

applywarp --ref=${FSLDIR}/data/standard/MNI152_T1_2mm --in=${i} --warp=${i}_step3 --out=${i}_step4

######################################################################

LINK: <http://fsl.fmrib.ox.ac.uk/fsl/fslwiki/FNIRT>

######################################################################

#### 5. Invert nonlinear warp (“step 4a”) ############################

## Invert the nonlinear warp in order to be able to back-project the ## MNI brain into subject space (this is the first part of “step 4” in ## the manuscript).

#

echo 5. invert nonlinear warp for subject ${i}

invwarp -w ${i}_step3.nii.gz -o ${i}_step5.nii.gz -r ${i}_step1.nii.gz

######################################################################

LINK: <http://fsl.fmrib.ox.ac.uk/fsl/fslwiki/FNIRT>

######################################################################

#### 6. Apply inverted nonlinear warp to labels (“step 4b”) ##########

## Apply inverted nonlinear warp to the MNI standard brain in order to ## back-project it back into subject space (this is the second part of ## “step 4” in the manuscript)

#

echo 6. apply inverted nonlinear warp to MNI label: MNI152_T1_1mm_brain_mask for subject ${i}

applywarp --ref=${i} --in=${FSLDIR}/data/standard/${mask} --warp=${i}_step5.nii.gz --out=${i}_step6 --interp=nn

######################################################################

LINK: <http://fsl.fmrib.ox.ac.uk/fsl/fslwiki/FNIRT>

######################################################################

#### 7. binarize brain extractions ###################################

## Binarize the back-projected MNI brain in order to use it for

## “punch-out” brain extraction (in the next step)

#

echo 7. creating binary brain mask for subject ${i}

fslmaths ${i}_step6.nii.gz -bin ${i}_optiBET_brain_mask

######################################################################

REFERENCE: Smith SM, Jenkinson M, Woolrich MW, Beckmann CF, Behrens TE, et al. (2004) Advances in functional and structural MR image analysis and implementation as FSL. NeuroImage 23 Suppl 1: S208-219.

LINK: <http://fsl.fmrib.ox.ac.uk/fsl/fslwiki/Fslutils>

######################################################################

#### 8. Punch-out mask from brain to do skull-stripping (“step 4c”) ##

## Take the binarized back-projected MNI brain and use it to “punch-

## out non-brain tissue from the subject’s original T1 image (this is the last part of “step 4” as described in the manuscript).

#

echo 8. creating brain extraction for subject ${i}

fslmaths ${i} -mas ${i}_optiBET_brain_mask ${i}_optiBET_brain

######################################################################

REFERENCE: Smith SM, Jenkinson M, Woolrich MW, Beckmann CF, Behrens TE, et al. (2004) Advances in functional and structural MR image analysis and implementation as FSL. NeuroImage 23 Suppl 1: S208-219.

LINK: <http://fsl.fmrib.ox.ac.uk/fsl/fslwiki/Fslutils>

######################################################################
